# Effect of the myostatin locus on muscle mass and intramuscular fat content in a cross between mouse lines selected for hypermuscularity

**DOI:** 10.1186/1471-2164-14-16

**Published:** 2013-01-16

**Authors:** Stefan Kärst, Eva M Strucken, Armin O Schmitt, Alexandra Weyrich, Fernando PM de Villena, Hyuna Yang, Gudrun A Brockmann

**Affiliations:** 1 Department for Crop and Animal Sciences, Breeding Biology and Molecular Genetics, Humboldt-Universität zu Berlin, Invalidenstraße 42, 10115, Berlin, Germany; 2 Department of Genetics, School of Medicine, University of North Carolina, 120 Mason Farm Road, Chapell Hill, NC, 27599-7264, USA; 3 Faculty of Science and Technology, Universitätsplatz 5 - piazza Università, 539100, Bozen-Bolzano, Italy

## Abstract

**Background:**

This study is aimed at the analysis of genetic and physiological effects of myostatin on economically relevant meat quality traits in a genetic background of high muscularity. For this purpose, we generated G_3_ populations of reciprocal crosses between the two hypermuscular mouse lines BMMI866, which carries a myostatin mutation and is lean, and BMMI806, which has high intramuscular and body fat content. To assess the relationship between muscle mass, body composition and muscle quality traits, we also analysed intramuscular fat content (IMF), water holding capacity (WHC), and additional physiological parameters in *M. quadriceps* and *M. longissimus* in 308 G_3_-animals.

**Results:**

We found that individuals with larger muscles have significantly lower total body fat (r = −0.28) and IMF (r = −0.64), and in females, a lower WHC (r = −0.35). In males, higher muscle mass was also significantly correlated with higher glycogen contents (r = 0.2) and lower carcass pH-values 24 hours after dissection (r = −0.19). Linkage analyses confirmed the influence of the myostatin mutation on higher lean mass (1.35 g), reduced body fat content (−1.15%), and lower IMF in *M. longissimus* (−0.13%) and *M. quadriceps* (−0.07%). No effect was found for WHC. A large proportion of variation of intramuscular fat content of the *M. longissimus* at the myostatin locus could be explained by sex (23%) and direction-of-cross effects (26%). The effects were higher in males (+0.41%). An additional locus with negative over-dominance effects on total fat mass (−0.55 g) was identified on chromosome 16 at 94 Mb (86–94 Mb) which concurs with fat related QTL in syntenic regions on SSC13 in pigs and BTA1 in cattle.

**Conclusion:**

The data shows QTL effects on mouse muscle that are similar to those previously observed in livestock, supporting the mouse model. New information from the mouse model helps to describe variation in meat quantity and quality, and thus contribute to research in livestock.

## Background

In livestock production, there is a high interest in controlling meat quantity and quality; knowledge about genes affecting muscle size and other meat properties can help breeders to select animals according to desired traits. Myostatin (*Mstn*, or growth and differentiation factor 8 - *Gdf*8) was first identified in knock-out mice as being a gene responsible for regulation of muscle growth [1]. However, the hypermuscular phenotype that originates from a hyperplasia and hypertrophy of muscle fibres was known long before its molecular-genetic background was elucidated [2]. In sheep, pigs and cattle, several mutations of myostatin or its promoter region have been identified as affecting muscle size [3-6]. Furthermore, myostatin also influences glucose metabolism and fat accumulation, as shown in knock-out mice that had smaller adipocytes [7] and did not develop obesity on a high-fat diet. These results suggested an altered metabolism for the utilization of lipids and glucose as energy fuel that prevented insulin resistance in mice [8,9]. A decreasing effect on intramuscular fat (IMF) content and carcass fat proportion was also described for the “double-muscled” phenotypes in cattle [10,11], which was later found to be caused by a myostatin mutation as well. However, different myostatin mutations can cause different degrees of hypermuscularity. The most extreme form of this phenotype in cattle is seen in the Belgian Blue, while different mutations with less extreme phenotypes were identified in other breeds [12].

Due to its large implication, not only on skeletal muscles, a lot of research has been undertaken on the effects of myostatin and its possible applications in health care and livestock production. The *myostatin*-gene, for instance, appears to be a promising candidate for the treatment of muscle dystrophy diseases [13,14], cardiac tissue regeneration or metabolic syndrome [15,16]. In livestock, efforts to manipulate the gene’s expression through immunization have been made for the purposes of higher lean mass production [17]. In accordance with this, modifiers of myostatin, such as follistatin (*Fst*), which inhibits myostatin, are currently under review as therapeutics [18,19].

In the present study, we generated G_3_-populations of reciprocal crosses between two hypermuscular Berlin Muscle Mouse Inbred (BMMI) lines to examine the genetic characteristics of myostatin and to find additional genes that could influence muscle growth and composition. The BMMI lines were hypermuscular as a result of long term selection that mirrors the selection process for high meat yield in livestock. The parental BMMI866 line originates from a population with the “compact” phenotype and therefore carries the known *Mstn*^*Cmpt-dl1Abc*^ mutation [20,21]. Although the BMMI806 line originates from the same founder population, it does not carry this mutation; BMMI806 animals display very high intramuscular fat contents, fat mass and fat proportion, especially in males [22]. Previously, genetic modifier regions for the effect of the myostatin *Mstn*^*Cmpt-dl1Abc*^ mutation on muscle mass have been identified on chromosomes 3, 5, 7, 11, 16, and X in a cross between Comp9 and CAST/Ei lines [23].

Given that the BMMI lines were originally developed as supporting genetic models for livestock research, we were particularly interested in myostatin effects on intramuscular fat content (IMF) and water holding capacity (WHC) on the genetic background of high muscularity. We also analysed the extent to which sex and the direction of the reciprocal cross impacted on the traits of interest. The latter could indicate parent-of-origin effects, where the impact on the phenotype can be different depending on the parent from which an allele was inherited. For example, the polar over-dominance caused by the ovine callipyge locus, where a hypermuscular phenotype only occurs if the mutated allele is inherited from the sire [24,25]. Parent-of-origin effects have also been described for body composition and fat-related traits in mice, pigs and cattle [26-29].

In addition to the relationship between muscle mass and meat quality traits, we were also interested in certain parameters of the muscle and whole body metabolism such as muscle glycogen and lactate contents, blood glucose levels, and the carcass pH-values. For this purpose, we present the correlations between these traits in the G_3_-population. The linkage study did not reveal genomic loci accounting for variation of those metabolic traits.

## Results and discussion

### Phenotypes

As shown in Table 1, significant differences were found between the two parental lines. Averaged over both sexes, the *Mstn*^*Cmpt-dl1Abc*^ mutant BMMI866 animals showed 42%, 42%, 99% and 94% higher values for body weight, lean mass, *M. longissimus* and *M. quadriceps* masses than the BMMI806, respectively. BMMI866 mice had 30% lower total fat percentage than BMMI806. The IMF contents of the *M. longissimus* and the *M. quadriceps* were 52% and 40% lower, respectively, as compared to the BMMI806 line. These data confirm the hypertrophic effect of the *Mstn*^*Cmpt-dl1Abc*^ mutation and its impact on fat accumulation [7]. Furthermore, fasting blood glucose levels of the BMMI866 line were 14% below the levels of BMMI806. The decreased glucose levels of BMMI866 mice support the model of a metabolic shift towards the utilization of glucose as energy fuel if myostatin is not fully functional, as shown by experiments in cell cultures [30]. Regarding differences between the sexes, male BMMI866 mice had lower carcass pH-values after 1 hour and female BMMI866 mice showed lower carcass pH-values after 24 hours *post-mortem* compared to BMMI806 (*p* < 0.05).

**Table 1:** Body traits of parental, F_1_ and G_3_ animals (means and standard deviations)

	**P**				**F1**		**G3**	
	**BMMI866**		**BMMI806**					
**Trait**	**Males**	**Females**	**Males**	**Females**	**Males**	**Females**	**Males**	**Females**
Body weight (g)	49.31 (2.36)***	40.35 (2.47)***	36.43 (4.69)	26.77 (2.16)	42.17 (3.44)^*aaa,bb*^	34.38 (2.12)^*aaa,bbb*^	44.98 (4.21)^*aaa,bbb*^	36.69 (4.63)^*aaa,bbb*^
Lean mass (g)	41.89 (2.43)***	33.79 (2.12)***	29.31 (2.29)	23.87 (1.99)	32.30 (2.72)^*aaa,bbb*^	29.04 (2.03)^*aaa,bbb*^	36.12 (4.03)^*aaa,bbb*^	30.61 (4.42)^*aaa,bbb*^
ML-Mass (g)	1.33 (0.13)***	1.08 (0.1)***	0.67 (0.08)	0.54 (0.05)	0.89 (0.09)^*aaa,bbb*^	0.75 (0.08)^*aaa,bbb*^	0.95 (0.22)^*aaa,bbb*^	0.85 (0.23)^*aaa,bbb*^
MQ-Mass (g)	0.84 (0.05)***	0.67 (0.05)***	0.44 (0.04)	0.34 (0.03)	0.55 (0.05)^*aaa,bbb*^	0.47 (0.03)^*aaa,bbb*^	0.61 (0.15)^*aaa,bbb*^	0.55 (0.15)^*aaa,bbb*^
Fat mass (g)	3.53 (1.67)	3.50 (1.35)	4.41 (1.46)	3.13 (1.19)	4.41 (0.98)^*a*^	3.76 (0.94)	5.00 (1.96)^*aa*^	2.88 (1.39)^*a*^
Fat mass (%)	7.77 (3.65)***	9.36 (3.49)	12.90 (3.52)	11.41 (3.47)	11.98 (2.35)^*aaa*^	11.40 (2.31)^*a*^	12.16 (4.50) ^*aaa*^	8.70 (4.31)^*b*^
IMF-ML (%)	1.17 (0.23)***	1.28 (0.23)***	2.64 (0.34)	2.45 (0.49)	1.89 (0.32)^*aaa,bbb*^	1.50 (0.2)^*a,bbb*^	1.69 (0.39)^*aaa,bbb*^	1.38 (0.33)^*bbb*^
IMF-MQ (%)	1.07 (0.15)***	1.11 (0.12)***	1.81 (0.30)	1.80 (0.29)	1.46 (0.15)^*aaa,bbb*^	1.38 (0.16)^*aaa,bbb*^	1.34 (0.21)^*aaa,bbb*^	1.22 (0.18)^*aa,bbb*^
WHC-ML (%)	1.00 (0.58)	1.03 (0.39)	1.09 (0.37)	1.01 (0.39)	0.90 (0.25)^*b*^	1.08 (0.35)	1.24 (0.64)^*aa*^	1.23 (0.41)^*a*^
WHC-MQ (%)	0.75 (0.21)	0.69 (0.17)	0.85 (0.33)	0.81 (0.26)	0.86 (0.17)^*a*^	0.95 (0.33)^*aa*^	0.93 (0.29)^*aa*^	0.93 (0.23)^*aaa,b*^
Glucose (mg/dL)	105 (24)*	88 (17)**	120 (26)	104 (17)	108 (14)	87 (12)^*bbb*^	106 (13)^*bb*^	90 (12)^*bbb*^
Glycogen (mg/g)	1.94 (0.75)	2.24 (1.51)	2.47 (0.76)	2.45 (1.38)	1.52 (0.52)^*b*^	1.73 (0.54)	1.37 (0.90)^*bb*^	1.18 (0.76)^*b*^
Lactate (mg/g)	0.068 (0.008)	0.091 (0.014)	0.064 (0.003)	0.070 (0.025)	0.035 (0.010)^*aa,bb*^	0.035 (0.012)^*a,b*^	0.048 (0.017)^*aa,bb*^	0.045 (0.013)^*aa,bb*^
pH 1 hour	6.67 (0.11)*	6.63 (0.08)	6.74 (0.09)	6.71 (0.12)	-	-	6.77 (0.15)^*aaa*^	6.73 (0.15)^*aa*^
pH 24 hours	6.16 (0.10)	6.15 (0.12)*	6.12 (0.12)	6.25 (0.12)	-	-	6.12 (0.11)	6.13 (0.09)^*bbb*^

Trait abbreviations: IMF = intramuscular fat content*,* WHC = water holding capacity (measured as ‘drip loss’ in percent of muscle weight), ML = *Musculus longissimus*, MQ = *Musculus quadriceps*, Glucose = blood glucose level. Phenotypes of the parental lines are compared with each other and the F_1_ and G_3_, separated for sex. Asterisks indicate significant differences between the parental lines; letters indicate significant differences between the F_1_ or G_3_ and BMMI866 (^*a*^) or BMMI806 (^*b*^) lines. Number of asterisks and letters show levels of significance (***^/aaa/bbb^*p* < 0.001, **^/aa/bb^*p* < 0.01, *^/a/b^*p* < 0.05).

Comparing the F_1_ and G_3_-population with the parental lines, we found intermediate values for body weight, lean mass and muscle masses. Dominance of the BMMI806 characteristics was indicated for total fat percentage in the F_1_ population. While the IMF values of both sexes of F_1_ animals and the males of the G_3_ population were intermediate, in female G_3_ animals, the measurements of total fat percentage and IMF of the *M. longissimus* were similar to the lower values of the BMMI866 line. Fasting blood glucose levels indicated a dominance of the BMMI866 line alleles, whereas the BMMI806 phenotype was found prevalent for the higher carcass pH-values for 1 hour *post mortem*.

### Correlations

In the G_3_-population, high positive correlations in males and females were observed between muscle mass and total lean mass (r > 0.79) and negative correlations with body fat percentage (r < −0.46) and IMF (r < −0.53; Table 2). In females, muscle mass was also negatively correlated with WHC in *M. quadriceps* (r = −35), whilst in males, no correlation was found. Furthermore, in males, higher muscle mass was associated with lower pH-values 24 hours post mortem (r < −0.16). Nevertheless, in both sexes negative correlations were evident between muscle glycogen contents and the pH-values of 1 and 24 hours *post mortem* (r < −0.15), and between WHC and the pH-value after 24 hours (r < −0.14). The muscle glycogen content was associated with the body fat content (r < −0.17), but was not correlated with the blood glucose levels; higher muscle glycogen contents were found in leaner animals.

**Table 2:** Spearman’s correlation coefficients between different traits in the G_3_ population

**Males**															
	**Body Weight (g)**	**Lean Mass (g)**	**ML-Mass (g)**	**MQ-Mass (g)**	**Fat Mass (g)**	**Fat Mass (%)**	**IMF-ML (%)**	**IMF-MQ (g)**	**Glucose (mg/dL)**	**WHC-ML (%)**	**WHC-MQ (%)**	**Glycogen (mg/g)**	**Lactate (mg/g)**	**pH, 1 hour**	**pH, 24 hours**
Body weight (g)		0.88***	0.66***	0.66***	0.12 **.**	−0.12 **.**	−0.31***	−0.23**	0.01	−0.01	−0.02	0.12	−0.02	−0.15*	−0.19**
Lean mass (g)	0.95***		0.80***	0.79***	−0.27***	−0.50***	−0.50***	−0.49***	−0.06	0.01	−0.04	0.15*	−0.06	−0.07	−0.17*
ML-Mass (g)	0.82***	0.87***		0.91***	−0.32***	−0.51***	−0.65***	−0.69***	−0.01	0.04	−0.04	0.20**	−0.02	−0.12	−0.19**
MQ-Mass (g)	0.82***	0.87***	0.96***		−0.28***	−0.46***	−0.66***	−0.64***	−0.03	0.05	−0.1	0.11	−0.04	−0.12.	−0.16*
Fat mass (g)	−0.10	−0.32***	−0.28**	−0.29**		0.96***	0.52***	0.62***	0.03	−0.01	−0.03	−0.13 **.**	0.09	−0.14*	−0.11
Fat mass (%)	−0.34***	−0.54***	−0.48***	−0.49***	0.96***		0.60***	0.70***	0.04	−0.02	−0.02	−0.17*	0.08	−0.1	−0.05
IMF-ML (%)	−0.51***	−0.60***	−0.64***	−0.66***	0.45***	0.56***		0.67***	0.05	0.05	0.03	−0.15*	−0.01	0.03	0.08
IMF-MQ (g)	−0.35***	−0.45***	−0.57***	−0.53***	0.37***	0.46***	0.52***		0.03	0.03	0.09	−0.12	−0.09	0	0.08
Glucose (mg/dL)	0.05	0.05	−0.08	−0.01	0.01	0.01	0.13	0.11		−0.01	0.01	0	−0.03	−0.05	−0.05
WHC-ML (%)	−0.16 **.**	−0.13	−0.15	−0.09	−0.16 **.**	−0.09	−0.05	0.03	0.15		0.35***	0.15*	−0.02	0.08	−0.21**
WHC-MQ (%)	−0.38***	−0.39***	−0.35***	−0.35***	0.04	0.13	0.09	0.1	0.04	0.42***		0.10	0.10	0.11	−0.14 **.**
Glycogen (mg/g)	0.09	0.10	0.13	0.12	−0.24*	−0.23*	−0.04	−0.02	0.14	0.05	0.03		−0.05	−0.16*	−0.27***
Lactate (mg/g)	0.04	0.06	0.10	0.08	−0.05	−0.07	−0.14	−0.20*	0.06	0.14	0.15	0.07		−0.17*	−0.15*
pH, 1 hours	0.05	0.07	−0.11	−0.12	0.01	−0.01	0.12	0.08	0.01	−0.02	−0.06	−0.20*	−0.17 **.**		0.18**
pH, 24 hours	0.03	0	−0.08	−0.07	−0.01	−0.01	0.04	0.01	0.01	−0.23*	−0.16 **.**	−0.31***	−0.23*	0.23*	
**Females**															

Trait abbreviations: IMF = intramuscular fat content*,* WHC = water holding capacity, measured as ‘drip loss’ in percent of muscle weight, ML = *M. longissimus*, MQ = *M. quadriceps*. Glucose = blood glucose level; Asterisks indicate the significance of correlations: *** *p* < 0.001, ** *p* < 0.01, * *p* < 0.05, **.***p* < 0.10.

The results regarding the carcass pH-value and WHC confirm studies in pigs, where a low pH-value negatively affects WHC and leads to high drip loss. The low WHC is likely the result of a developing acidic environment that affects the proteolysis of cell scaffold proteins [31,32]. In turn, the acidic environment is caused by high intramuscular glycogen contents that promote the development of high lactate levels via glycolysis [31]. The negative correlation between muscle glycogen and body fat content as well as IMF of the *M. longissimus* in males supports the model of a metabolic shift towards glycolysis. The non-significant correlation with fasting blood glucose levels is likely due to the short fasting period of two hours before measurements leading to a high variability.

### QTL analysis

**Figure 1:**
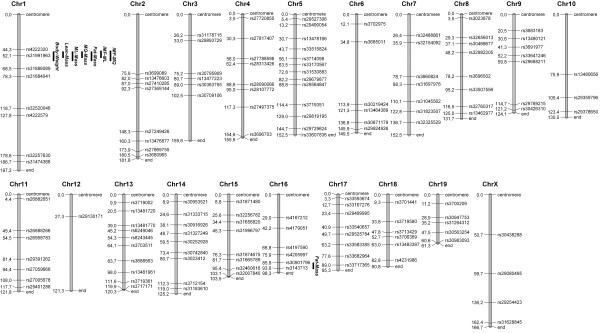
**MapChart plot of 138 reference single nucleotide polymorphisms used in this study.** Positions are given in Mb (NCBI Build 37). Bars indicate LOD support intervals of identified significant QTL.

**Figure 2:**
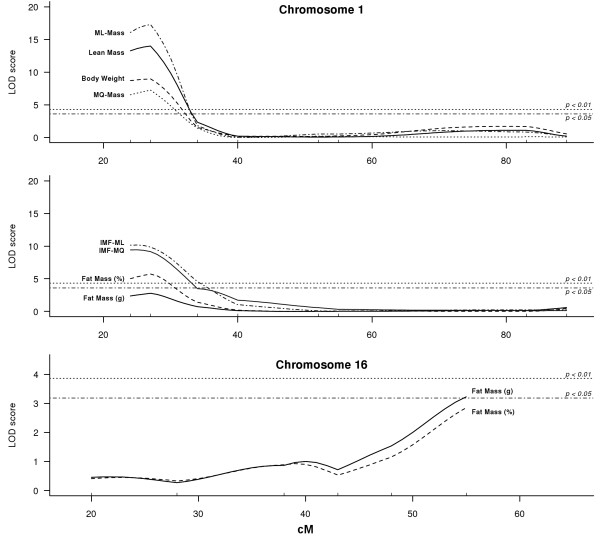
**QTL scans for different traits on chromosome 1 and 16.** Sex and direction-of-cross were used as additive covariates and genome-wide significance thresholds of *p* < 0.05 and *p* < 0.01.

**Figure 3:**
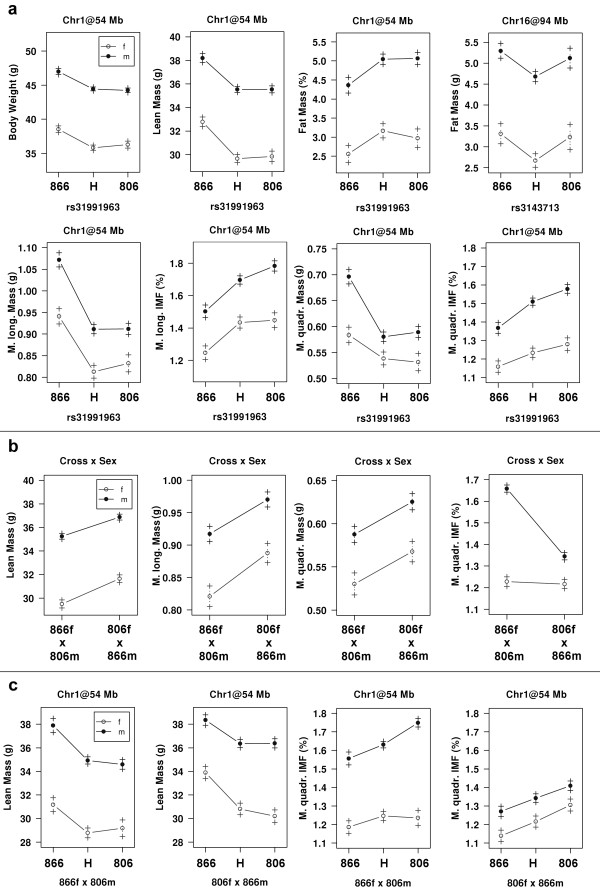
**Effect plots showing means and standard errors of the three genotype-classes at significant QTL positions. a**) QTL significant under 95% and **b**) sex and direction-of-cross effects; **c**) directions-of-cross effects for the three genotypes for lean mass and IMF of the *M. quadriceps***;** 866 = homozygous BMMI866-allele, 806 = homozygous BMMI806-allele, H = heterozygous animals; 806f x 866m = G_3_-animals originating from mating a female BMMI806 and a male BMMI866, 866f x 806m G_3_-animals originating from mating a female BMMI866 and a male BMMI806 in the parental generations.

Due to the relative small number of animals in our G_3_-population, we could only detect regions with larger effects. Two genome-wide significant QTL were identified (Figure 1). The first one was located on chromosome (Chr) 1 at 54 Mb (47–59 Mb) in a region that contains the previously described myostatin *Mstn*^*Cmpt-dl1Abc*^ mutation, located at 53 Mb (Figure 2). In the G_3_-population, the BMMI866 allele at this locus caused additive effects of 1.25 g for body weight and 1.35 g for lean mass. It also increased the masses of the *M. longissimus* and *M. quadriceps* muscles by 0.07 g and 0.04 g, respectively (Table 3). The body fat percentage was decreased by the BMMI866 allele by 1.15 points. A similar effect was observed for IMF where the BMMI866 allele had a decreasing effect of 0.13 and 0.07 percentage points for IMF values of the *M. longissimus* and *M. quadriceps*, respectively (Figure 3a, Table 3).

**Table 3:** **QTL, sex and direction-of-cross effects (*p*** **< 0.001) in the G**_**3**_**population at age of 10 weeks**

**Trait**	**QTL/Effect**^**1**^	**Chr**	**Mb**^**2**^	**CI**^**3**^	**Marker**^**4**^	**LOD**^**5**^	**a (s.e.)**^**6**^	**d (s.e.)**^**6**^	**% G_3_ var^7^**
Body weight (g)	sex					83.01	8.43 (0.31)		68.35
	pgm					6.44	1.67 (0.3)		2.81
	1@27.0	1	54	47-59	rs31991963	8.93	−1.25 (0.21)	−1.03 (0.31)	3.97
Lean mass (g)	sex					62.43	5.74 (0.27)		55.22
	pgm					7.78	1.59 (0.26)		4.42
	1@27.0	1	54	47-55	rs31991963	13.91	−1.35 (0.18)	−1.2 (0.26)	8.27
ML-Mass (g)	sex					14.30	0.1 (0.01)		15.31
	pgm					3.43	0.04 (0.01)		3.38
	1@27.0	1	54	47-59	rs31991963	17.26	−0.07 (0.01)	−0.07 (0.01)	18.90
MQ-Mass (g)	sex					8.68	0.07 (0.01)		10.86
	pgm					2.50	0.03 (0.01)		2.98
	1@27.0	1	54	47-55	rs31991963	7.17	−0.04 (0.01)	−0.04 (0.01)	8.88
Fat mass (g)	sex					35.31	1.98 (0.15)		40.23
	16@55.0	16	94	86-94	rs3143713	3.57	−0.07 (0.12)	−0.55 (0.15)	3.16
Fat mass (%)	sex					15.49	2.97 (0.36)		18.85
	1@27.0		54	47-59	rs31991963	6.32	1.03 (0.24)	1.15 (0.35)	7.17
IMF-ML (%)	sex					20.04	0.28 (0.03)		22.55
	1@27.0	1	54	47-55	rs31991963	10.15	0.13 (0.02)	0.06 (0.03)	10.56
IMF-MQ (%)	sex					50.46	0.41 (0.02)		37.56
	pgm					39.19	−0.01 (0.03)		26.50
	1@26.0	1	51	47-59	rs31991963	10.05	0.07 (0.01)	0.00 (0.02)	5.36
	sex:pgm					16.08	−0.29 (0.03)		9.00

^1^ QTL positions are given as chromosome number, @, and the genetic distance from the centromer in cM.

^2^ Most likely chromosomal location given as Mb-position.

^3^ 1-LOD support interval in Mb.

^4^ Marker closest to the chromosomal position with the highest LOD score.

^5^ LOD scores from the full model estimations.

^6^ additive (*a*) and dominance (*d*) effect and their standard errors (s.e.) determined with the non-transformed raw trait values, therefore given in the respective unit; the direction of *a* and *d*, respectively given as the effect of the BMMI866-allele.

^7^ G_3_ phenotypic variance (%) explained by the QTL; QTL effect given as the reduction of the residual sum of squares fitting 1 *vs.* 0 QTL.

An additional QTL was identified on Chr16 at 94 Mb (86–94 Mb; Figure 2) that decreased the total fat mass by 0.55 g in heterozygous animals (Figure 3a, Table 3). This is a negative heterosis effect where heterozygous animals have significantly lower t values than either homozygous class**.** This significant negative dominance for fat mass at the Chr. 16 QTL is interesting because it is in contrast to the phenotypic observation that male F1 and G3 individuals tended to have a higher fat mass than the mean of to the parental lines (Table 1). The general positive heterosis of the offspring lines points to other loci with effects on fat mass that could not be detected in this study. The QTL on chromosome 16 showed no linkage with IMF, which indicates a specific role in adipose tissue for this region in mice. An interaction between the Chr1 and the Chr16 locus was not observed, and thus our data provides evidence that the region on Chr16 is an independent regulator of total fat mass. Up to now only one marker in that region, the D16Mit51 at 93 Mb, was proposed provisionally by Srivastava *et al.* (2006) as causative for variation in total fat mass in mice.

The QTL for fat mass on Chr16 is located within a region from 75–98 Mb that is syntenic with a region in pigs on *Sus scrofa* chromosome (SSC) 13 (129–145 Mb). According to the animal genome database (animalgenome.org) this region contains QTL for marbling, back fat and for IMF [32-35]. Dominance effects for fat mass, as they were found in our study, were not significant for the syntenic region in pigs, however a positive dominance was found for ham weight [36-38]. Thus, this syntenic locus could possibly have a negative dominance effect on fat mass because muscle weight and fat weight are often negatively correlated. Furthermore, similar effects were observed for the syntenic region in cattle. In cattle, this region is divided into two parts which reside on chromosome 1 (BTA1) between 0–23 Mb and 141–155 Mb. Within those two regions on BTA1, fat related QTL were mapped for milk fat yield, marbling and fat thickness at the 12th rib [39-42]. A positive but non-significant dominance effect on adjusted subcutaneous fat thickness was reported in cattle [40]. Finally, the syntenic region in the human genome resides on chromosome 21 at 15–43 Mb. The Human Obesity Gene Map counts four obesity-associated loci in this syntenic region [43,44]. Assuming that the same gene is underlying the variation across species, further refinement of the murine Chr16 regions seems promising and our study adds to the confirmation of mice as a model animal to study meat quality traits in livestock or muscular disorders in humans.

As we did not find any other loci affecting muscle mass apart from the myostatin locus, we assume that several or many small effect alleles that contribute to these traits and that have been shown in other crosses [45] are hidden in our study behind the strong effect of myostatin, in particular in the hypermuscular line BMMI806. Another reason could be that the statistical power was not sufficient in the examined population.

### Effects of sex and direction-of-cross

As shown in Table 3, sex affected all traits for all identified QTL. Males significantly increased body weight, lean mass, and the masses of the *M. longissimus* and *M. quadriceps* by 8.43 g, 5.74 g, 0.10 g and 0.07 g, respectively. In males, the total fat mass and the total fat percentage were also raised by 1.98 g and 2.97 points, respectively. Sex also affected IMF values of the two examined muscles, which were increased by 0.28 points and 0.41 points for the *M. longissimus* and the *M. quadriceps* in males, respectively. The increase in the lean mass related traits was expected as a sex difference; however, the increase in fat mass percentage in male mice was a new observation. An explanation could be that male BMMI806 mice display very high fat values (Table 1) and the high-fat phenotype in G_3_-males is thought to be inherited by the BMMI806 alleles of both QTLs.

Interestingly, for the myostatin locus, we also found significant effects of the direction-of-cross and an interaction between sex and the direction-of-cross on IMF of the *M. quadriceps* (LOD = 13.62). Animals derived from mating a female BMMI806 with a male BMMI866 showed higher body weight, lean mass and muscle mass at the Chr1 locus in comparison to animals of the reciprocal cross. While total fat mass, fat percentage and IMF of the *M. longissimus* were not significantly affected, we observed a large increase of IMF in the *M. quadriceps* by 0.29 points in male G_3_-animals that were derived from a mating between a female BMMI866 and a male BMMI806 (Figure 3b,c). This means that in male G_3_-animals descending from a BMMI806 grand-grandfather, myostatin expressed less of its IMF decreasing effect. Furthermore, the effects of myostatin were generally larger in G_3_-animals descending from a male BMMI866 parent of the initial cross.

As a likely result of the recent selection and inbreeding process, SNP data of the mitochondrial DNA and the Y-chromosome did not differ between the two parental lines. Therefore, we assumed that mitochondria and Y-chromosomes did not account for trait differences between reciprocal crosses. Nevertheless, we cannot completely exclude hidden mutations derived during the breeding history. Such hidden mutations do not play a role for mitochondria, but might affect Y-chromosomal effects. However, so far, no Y-chromosomal effects on traits that we analysed in this study have been identified. Therefore, the phenotypic differences between the reciprocal crosses are likely parent-of-origin effects.

The high IMF values for the QTL on Chr1, for G_3_-males of the cross between a BMMI866 female and a BMMI806 male, were very similar to males of the parental line BMMI806. This was not observed in the reciprocal cross and therefore, might result from parent-of-origin effects. Since evidence is growing that parent-of-origin effects could exist over several generations [46] our findings suggest epigenetic patterns, which alter the gene expression of the chromosomes according to the ancestor they were inherited from. Whether the myostatin locus alone underlies genetic imprinting, or additional loci that did not directly affect the analysed traits, still has to be tested in a genome-wide screen.

Recently, we have identified bidirectional parent-of-origin effects for muscle glycogen content and glycolytic potential as well as body weight in a population between two other hypermuscular mouse lines BFMI806 × BMMI816 [47]. The imprinted loci were not discovered by a genome-wide QTL search directly for additive and dominance effects.

In sheep with the callipyge mutation, the callipyge phenotype was not expressed in homozygous mutated animals [24,25]. It was suggested that the maternal inactivation of the locus also negatively affected the paternal expression, but a paternally inherited mutated locus escapes the inactivation in the presence of a maternally inherited wild-type allele and leads to the callipygous phenotype. Phenomenon like this have their molecular origin in a DNA methylation ‘reset’ that occurs during gametogenesis, the fusion of oocyte and spermatocyte in the zygote and the following early embryogenesis [48,49]. As seen in the callipyge sheep example, small genetic differences can affect the reprogramming processes and lead to different gene expression patterns and phenotypes.

## Conclusions

The strong effects of the myostatin mutation *Mstn*^*Cmpt-dl1Abc*^ on high muscle and low fat mass were confirmed, even in a background of high muscularity in a cross between two hypermuscular mouse lines. The data provides evidence for sex and very likely parent-of-origin effects modifying the direct effects of the myostatin locus on muscle mass and IMF in *M. quadriceps*. However, further tests are necessary to confirm imprinting effects. In addition to the myostatin locus, we have shown that a region on Chr 16 affecting fat mass is conserved between mice, pig and cattle showing effects on body fat, marbling and milk fat. Furthermore, many reported observations and correlations of the *post mortem* physiology of livestock were reproduced in our mice.

The results obtained in this study help to justify the mouse model to study economically relevant livestock traits. New information from the mouse model helps to describe variation in meat quantity and quality and thus supports research in livestock.

## Methods

### Animals

The Berlin Muscle Mouse population had been long term selected for high body weight and high muscle mass to reflect the selective mechanisms in livestock breeding. Founder animals of the Berlin Muscle Mouse (BMM) population were originally purchased in several pet shops in Berlin, Germany. The selection process comprised several distinct phases. The beginning of the selection process constituted a phase of 23 generations of selection for high protein content of the carcass at the age of 60 days. Protein content was determined by chemical analyses. In a second phase, mice were selected for high body weight and low fat content at 42 days for 10 generations. Afterwards, mice were monitored for high muscularity by palpating and mice with highest muscularity on a scale between 1 and 5 were selected for the next generation. As a result of selection and likely the occurrence of natural mutation(s) over 25 generations, a mouse population with a high muscular phenotype had been generated. A high compact sub-line was perpetuated through random mating of selected animals [21]. Sequencing of the myostatin gene in this line revealed a 12 bp deletion [20] leading to a loss of function. Scale and weight based selection continued for another 28 generations. After 86 generations of selection, full-sibs with distinct phenotypes were mated. These founder animals became the basis of seven Berlin Muscle Mouse inbred lines (BMMI). Four lines display the *Mstn*^*Cmpt-dl1Abc*^ mutation and three are wild type. In this study we used the lines BMMI866, which carries the described *Mstn*^*Cmpt-dl1Abc*^ mutation, and BMMI806, which is hypermuscular but wild type for myostatin and shows high IMF. At the time of setting up the crossbred experiment, the lines were in generation 20 (BMMI866) and 21 (BMMI806) of inbreeding.

### Pedigree structure

Two pairs of full sibs of the Berlin Muscle Mouse inbred lines BMMI866 and BMMI806 were crossed reciprocally to generate F1, F2 and G_3_-intercross populations. F_2_-animals within every direction-of-cross were randomly mated under avoidance of sibling-mating [50], to generate 308 G_3_-animals. Altogether, there were 195 males and 113 females. From these 308 animals, 97 males and 53 females came from the cross between a female BMMI866 and a male BMMI806 mouse, while the reciprocal cross between a male BMMI866 and a female BMMI806 mouse consisted of 98 males and 60 females. Using this design, we would expect different mitochondria and Y-chromosomes segregation in the reciprocal crosses. Since high-density SNP data of the inbred strains BMMI866 and BMMI806 provided evidence for no variation in both mitochondria and Y-chromosomes, we assume that the two parental mouse strains have the same mitochondria and Y-chromosomes as a result of their breeding history (See also under Genotyping).

### Husbandry and feeding conditions

The mice were treated in accordance to, and all experimental protocols were approved by, the German Animal Welfare Authorities (approval no. G0405/08). The animals were maintained under conventional conditions at 22 ± 2°C and controlled lightning with a 12:12 hour light:dark cycle. They were kept in groups of two to four animals of the same sex per Macrolon cage and given *ad libitum* access to food and water. Until the age of 70 days, the animals were fed a standard breeding diet (‘Altromin standard breeding diet no. 1314 TPF’, Lage, Germany). This diet contained 27.0% crude protein, 5.0% crude fat, 4.5% crude fibre, 6.5% crude ash, 50.5% nitrogen free extract (starch and sugar), vitamins, trace elements and minerals (2988 kcal/kg metabolizable energy; thereof 27.0% energy from proteins, 13.0% from fat and 60.0% from carbohydrates).

### Phenotypic measures

After a fasting period of two hours, 71 day-old mice were anaesthetised under isoflurane and decapitated. The *Musculus longissimus* (ML) and *Musculus quadriceps* (MQ) were dissected and weighed. The summed muscle weights of the left and right *M. longissimus* and the left and right *M. quadriceps* were recorded as muscle mass (MM). The right muscles were immediately frozen in liquid nitrogen and subsequently stored at −80°C. The left muscles were cooled down to 6°C for one hour, subsequently frozen at −20°C and stored at the same temperature until WHC and IMF were measured. Carcasses were stored at 6°C and pH-values were taken within the *M. biceps femoris* at one and 24 hours *post mortem* (ebro PHT 810, Ingolstadt, Germany). For the determination of WHC, frozen muscles were thawed and stored at 6°C for 24 hours. Muscles were then centrifuged for 60 sec at 604 × g in invitek® 1.5 ml receiver tubes with filter inlays to collect the tissue fluid that was not held from the muscle (Eppendorf Minispin, Hamburg, Germany; Invitek, Berlin, Germany). The ratio of lost tissue fluid to tissue mass was designated as ‘drip loss’. IMF was measured as percentage of muscle weight using nuclear magnetic resonance technology (SMART Trac System, CEM, Kamp-Lintfort, Germany) [51]. Body weights were recorded weekly. At ten weeks, total fat and lean masses were measured by quantitative magnetic resonance (QMR) analysis, using the EchoMRI whole body composition analyser (Echo Medical Systems, Houston, Texas, USA) [22,52]. Body fat percentage is the percentage of total fat mass calculated from the sum of total fat and lean mass. Blood glucose levels were measured after two hours of fasting, prior to dissection at ten weeks (Bayer ‘Contour’, Leverkusen, Germany). Muscle glycogen content was determined colorimetrically in the right *M. longissimus* (GOD/PAP method ‘Glucose liquicolor’ by Human, Wiesbaden, Germany) as suggested by Barham and Trinder (1972). Lactate contents were determined colorimetrically in the right *M. longissimus* using the Lactate Assay Kit by Techung Lee [53].

### Genotyping

Parental BMMI lines were genotyped with the Mouse-Diversity-Array [54] comprising 623,124 single-nucleotide polymorphisms (SNPs). The SNP information provided evidence for allele fixation of 98.1% and 98.3% in lines BMMI866 and BMMI806 after 20 and 21 generations of inbreeding, respectively. Both lines differed from each other by 4.8% at the SNP level. SNPs of the mitochondria and the Y-chromosome were not different between the lines. Using the information on diverse genomic regions between BMMI806 and BMMI866, 138 informative SNP markers covering all chromosomes (except Y) in an average distance of 24.8 Mb were selected for genotyping the parents, F_2_ and the G_3_-animals (Figure 1). Regions larger than 10 Mb that did not differ in SNP-alleles between parental lines could not be included into the linkage analysis. We assume that these regions have the same ancestral origin as a result of the breeding history. Genotyping was done at KBiosciences (Hoddesdon, U.K.). The physical map was converted into the genetic map using the ‘Mouse Map Converter’ software from The Jackson Laboratory [55].

### QTL and statistical analyses

For the QTL analysis, 308 G_3_-animals were used. Analyses for single QTL detection and detection of interacting QTL were performed using R/qtl [56]. Unlike F_2_, the G_3_-animals may be unequally related to each other. Ignoring the unequal relatedness may result in a serious inflation of false positive rates. Therefore, we calculated environmental residuals for both sexes and each of the two crosses and corrected for litter size and genomic kinship. This part of the analysis was carried out with a Genome-wide Rapid Analysis using Mixed Models And Mixed Models and Regression (GRAMMAR) as implemented in GenABLE [57-60]. The environmental residuals were used to generate corrected phenotypes, which were applied in the actual QTL analysis. Direction-of-cross and sex were included as additive covariates in the model and used as interactive covariates to test their effects on QTL. All phenotypes were log-transformed to obtain normal distribution. Trait-specific significance thresholds were estimated with 1000 permutations [61]. On average, the thresholds for genome-wide significance and suggestive linkage results were LOD = 3.59 (*p* < 0.05) and LOD = 3.19 (*p* < 0.10), respectively. Genome-wide significant QTL (*p* < 0.05) were included into a mixed model for each trait to calculate the respective trait variance in the G_3_-population. Factors being significant under *p* < 0.001 were kept in the model. The genotype of the myostatin locus was accounted for by including it as a covariate in scans for additional QTL. Basic statistics were performed using the SAS software package (SAS System for Windows, Release 9.2). QTL support intervals were determined by bootstrapping as implemented in R/qtl [60].

## Competing interests

The authors declare that they have no competing interests.

## Authors’ contributions

SK conceived of the study, carried out the data collection, molecular genetic studies, statistical analysis of the data and drafted the manuscript. EMS and AOS participated in the statistical analysis, particularly in correcting the data for population stratification and estimating heterozygosity, respectively. AW carried out lactate and glucose measurements, FPMV and HY provided the data of the mouse diversity array (MDA). GAB participated in conceiving of the study, its design and coordination and helped drafting the manuscript. All authors read and approved the final manuscript.
